# Bariatric metabolic surgery and cancer risk: Target trial emulation using iterative time distribution matching

**DOI:** 10.17305/bb.2025.12842

**Published:** 2025-11-11

**Authors:** Jazeel Abdulmajeed, Zumin Shi, Manar E Abdel-Rahman, Fakhar Shahid, Mohammed F Alam, Mashael Al-Shafai, Muhammad E H Chowdhury, Abdullah Shaito, Adedayo A Onitilo, Suhail A Doi

**Affiliations:** 1 Department of Population Medicine, College of Medicine, QU Health, Qatar University, Doha, Qatar; 2 Business and Health Intelligence Department, Primary Health Care Corporation, Doha, Qatar; 3 Department of Nutrition Sciences, College of Health Sciences, QU Health, Qatar University, Doha, Qatar; 4 Department of Public Health, College of Health Sciences, QU Health, Qatar University, Doha, Qatar; 5 Department of Bariatric and Metabolic Surgery, Hamad Medical Corporation, Doha, Qatar; 6 Department of Biomedical Sciences, College of Health Sciences, QU Health, Qatar University, Doha, Qatar; 7 Biomedical Research Center (BRC), QU Health, Qatar University, Doha, Qatar; 8 Department of Electrical Engineering, Qatar University, Doha, Qatar; 9 Marshfield Clinic Health System, Inc. Marshfield Medical Center, Marshfield, WI, USA; 10 Wisconsin NCI Community Oncology Research Program (WiNCORP), University of Wisconsin, Madison, WI, USA

**Keywords:** Cancer, bariatric metabolic surgery, immortal time bias, iterative time distribution matching, observational study

## Abstract

Bariatric metabolic surgery (BMS) is a common intervention for severe obesity, yet its effects on cancer risk remain unclear. Observational studies and meta-analyses yield inconsistent findings, while randomized controlled trials often lack adequate follow-up to evaluate cancer outcomes. This study aims to emulate a target trial using observational data, employing a transparent and robust methodology to address this issue. We constructed a large retrospective cohort of adults with obesity in Qatar using electronic medical records from the public health system, with data available from 2018. We developed and applied iterative time distribution matching (ITDM) which is an iterative version of prescription time distribution matching (PTDM) as an improved approach to mitigate immortal time bias. This adaptation facilitated the alignment of time-zero (T_0_) between BMS recipients and non-recipients. Subsequently, we applied a Cox proportional hazards regression model, controlling for confounders and prognostic covariates, for data analysis. The final study cohort comprised 124,780 individuals aged 30 years and older, including 1465 who underwent BMS and 1583 who developed cancer during the follow-up period. The median follow-up duration was 7.79 years (IQR: 4.89–10.85). In the confounder- and prognostic covariate-adjusted Cox model, BMS was associated with a reduced hazard of cancer (HR 0.49, 95% CI 0.31 to 0.76). Given potential residual confounding and the limited outcome data, these findings provide preliminary evidence of a protective association and should be interpreted cautiously. This approach emphasizes transparency in trial emulation design, and future studies should focus on specific cancer types and long-term outcomes as additional data become available.

## Introduction

Obesity is a well-established risk factor for numerous chronic diseases, including type 2 diabetes, cardiovascular disease, and cancer [1–3]. Several biological mechanisms linking obesity to cancer initiation and progression have been identified [3, 4]. It is estimated that approximately 3.6% of all newly diagnosed cancers worldwide, and 13% of obesity-related cancers in adults aged 30 years or older, are potentially linked to an elevated BMI [5, 6]. Bariatric metabolic surgery (BMS) is increasingly utilized to address the consequences of obesity, with accumulating long-term evidence on its efficacy and safety over the past 25 years. However, its impact on cancer risk remains uncertain. A range of observational studies have explored the association between BMS for severe obesity and cancer risk, but findings are inconsistent. Some studies indicate that patients undergoing BMS experience a significantly reduced risk of developing cancer [7, 8], while others report no such reduction, particularly among men, the elderly, or specific cancers, notably upper gastrointestinal cancers [9–17]. Meta-analyses of these observational data have also yielded conflicting results [18, 19]. Over the past 15 years, at least 13 randomized controlled trials (RCTs) have compared BMS with lifestyle and medical therapies for type 2 diabetes, demonstrating that BMS leads to significantly greater short- to mid-term improvements in glycemic control, disease remission, cardiovascular risk factors, and chronic kidney disease [20]. However, these RCTs have not adequately addressed cancer risk due to limitations such as small sample sizes and insufficient duration to detect differences in critical outcomes, including cancer and cardiovascular disease. Furthermore, variations in the types of non-surgical treatments (lifestyle, medications, exercise) and adherence to these programs across studies complicate the interpretation of results.

Given the ongoing uncertainty, one potential solution is to enhance the observational design to improve its reliability regarding causality by emulating a target trial’s synchronization of eligibility and assignment at time-zero (T_0_). This approach is especially feasible and valuable due to the availability of extensive databases. An attempt to emulate such a trial was made in 2022 [21], but it did not achieve effective synchronization of assignment (to BMS or not) with eligibility at T_0_ of follow-up (see Discussion). To optimize this design, the emulated target trial should estimate the joint effect of the operative and postoperative components [22] by randomly assigning individuals to either (1) undergo BMS and postoperative monitoring or (2) receive no surgery during follow-up. Against this backdrop, the objective of the present study was to investigate the association between BMS and the incidence of any cancer in adults, within the framework of an appropriately emulated target trial where eligibility and assignment to the intervention could be considered synchronized at T_0_ based on the employed methodologies.

## Materials and methods

### Study design and setting

This study was designed to emulate a target trial using observational data to estimate the causal effect of BMS on cancer risk. Patient data were obtained from the Business and Health Intelligence (BHI) department at the Primary Health Care Corporation (PHCC) in Qatar. The electronic medical record system (Cerner Millennium) was established in 2016 and became fully operational by 2018. This platform is shared among all public health providers, including secondary and tertiary care centers such as Hamad Medical Corporation (HMC) and the National Center for Cancer Care and Research (NCCR).

### Study population and variables

Adults who interacted with the system between 2018 and 2024 were eligible if they had at least one obesity-related BMI measurement (≥30 kg/m^2^) before the age of 40, as we could not ascertain a more specific date for the obesity diagnosis. Although BMI is an imperfect surrogate for adiposity and cancer-relevant biology (as it does not account for fat distribution or body composition and may misclassify risk across age and ethnic groups), we believe, as suggested in a recent study [23], that BMI can serve as a useful screening tool for patients with potential obesity. Key variables included demographics (age, gender, nationality), clinical diagnoses (cancer and other obesity-related complications), anthropometric measures (BMI records), details of BMS, and relevant dates (date of birth, diagnoses, BMS, last healthcare encounter, and death). We assigned the following dates: the date participants reached age 30 as the date of origin, the date of BMS for those who underwent the surgery, and the exit date defined as the date of cancer diagnosis, date of death, last recorded healthcare encounter, or December 31, 2024, whichever occurred first. We then excluded individuals following a stepwise approach as follows:

*Individuals with cancer diagnoses prior to age 30 were excluded.*

*Individuals with an exit date before age 30 were excluded.*

*Individuals who underwent BMS prior to age 30 were excluded.*

*Individuals who received BMS after a cancer diagnosis were excluded.*

This process resulted in a cohort of patients who had no cancer diagnoses and did not undergo BMS until age 30, although they may have undergone BMS after this age.

### Emulating a target trial

The causal question investigated whether BMS reduces the incidence of cancer diagnoses during follow-up. A pragmatic trial would synchronize the initiation of the two treatment strategies (BMS or no BMS) with eligibility criteria (adults with obesity but without cancer at age 30) at T_0_ of follow-up. This synchronization of eligibility and treatment assignment at T_0_ is a fundamental principle of study design that is inherent in randomized trials (Table S1, emulation protocol).

To emulate this target trial using observational data and mitigate biases such as immortal time, lead time, and survivor bias, it was essential to avoid misclassifying follow-up time. Individuals could continuously meet the eligibility criteria after age 30, which meant they could qualify multiple times as T_0_, whether receiving BMS or not [24]. The literature describes several strategies to ensure accurate follow-up time classification, including prescription time distribution matching (PTDM), sequential Cox models, time-dependent Cox models, and marginal structural Cox models [25]. We identified PTDM as a straightforward and transparent approach that requires fewer analytical assumptions to address immortal time bias. However, PTDM has been reported to introduce residual bias due to its single-iteration T_0_ assignment, which may subsequently assign T_0_ after the end of follow-up for some individuals, leading to a final time distribution in the analysis that is less comparable across groups. To address this, we randomly distributed the immortal time in the surgery group (the time from age 30 to the date of BMS) to the BMS non-recipients over multiple iterations.

Similar to PTDM [26, 27], the process begins by designating the treatment initiation time (the end of the immortal time period) as T_0_ for BMS recipients. Next, we list the immortal time for all BMS recipients (from age 30 to T_0_) and randomly assign one of these time periods (with replacement) as the immortal time for each BMS non-recipient, thus designating their T_0_. This process is repeated multiple times, creating several columns of potential T_0_ values for each BMS non-recipient. Ineligible row values (beyond the study exit date) are marked as missing, and the maximum T_0_ values across the first, first two, first three iterations, and so on, are selected to establish a sequence of maximum T_0_ values based on the number of iterations. The final mT_0_ for BMS non-recipients corresponds to the iteration number, *k*, at which the overall Kolmogorov–Smirnov *D* value is minimized. This was visualized using a transition plot that illustrates the overall Kolmogorov–Smirnov *D* value comparing the distribution between groups against the iteration number (see Figure S1). This finding was further corroborated using cumulative distribution plots (distplot in Stata; see Figure S2). We named this new method the iterative time distribution matching (ITDM) method, which retains the core process of PTDM while enhancing alignment of the time distribution prior to analysis and minimizing drop-outs due to invalid T_0_ assignments.

### Confounder selection

A directed acyclic graph (DAG) was constructed using dagitty.net to inform the selection of a minimal adjustment set. The factors considered included Qatari nationality, gender, type 2 diabetes at origin, Class III obesity (BMI ≥ 40 kg/m^2^), age at entry to risk [28], and smoking status. These factors are prognostic for cancer [29] and are associated with selection for BMS (see Figure S3), forming the minimum adjustment set. It was assumed that any selection bias potentially introduced by informative censoring would be mitigated by adjusting for covariates influencing selection into the intervention groups (see Figure S3). Weight loss trajectory and metabolic remission were considered mediators of the BMS-cancer effect in the DAG.

### Ethical statement

This study was reviewed and approved by the Institutional Review Board (IRB) of the PHCC, reference number BUHOOTH-D-24-00045. The Qatar University IRB (QU-IRB) granted a review exemption, reference number QU-IRB 072/2025-EM. The study utilized de-identified data from a population health repository, ensuring that participant confidentiality and privacy are maintained in accordance with ethical guidelines and institutional policies. Informed consent was not required, as the study involved secondary data analysis without direct participant interaction.

### Statistical analysis

The ITDM algorithm’s iterative nature can result in slight variations in the final inclusion set across different computers, even when identical seed values are employed. Consequently, we executed multiple iterations with various seed values (see code in Supplemental data) until we observed the maximal inclusion of participants with failures, subsequently saving that dataset for final analysis. We conducted a descriptive comparison of the original and ITDM cohorts. A descriptive survival analysis of the BMS non-recipient cohort (excluding individuals with cancer prior to age 30) was performed to assess cancer incidence (per 1000 person-years) and cumulative survival among those who did not undergo BMS. These estimates were reported across age bands (0–5, 5–10, 10–15, and 15+ years post age 30) for cancer incidence and at 5, 10, and 15 years from age 30 for cumulative cancer-free survival.

A stratified Cox proportional hazards regression analysis was performed on the ITDM cohort, stratified by gender and smoking status, and adjusted for age at entry [28] and other covariates identified through the DAG. It is crucial to note that the 10-iteration sequence was required to create the ITDM cohort for analysis only once, due to the relatively large dataset. In contrast, smaller datasets necessitate multiple runs of the 10-iteration sequence until the optimal transition plot is achieved, given the increased variability in alignment per run. The results from the stratified Cox model estimated the causal relationship between BMS and cancer risk, presenting findings as hazard ratios (HRs). The Cox model’s time origin was established at age 30, thereby utilizing age as the time scale, with entry to the risk set at T_0_. This analysis adhered to protocol, with censoring performed at the last recorded encounter in the system, providing a valid estimate under the assumption of non-informative censoring [30]. The DAG indicates that adjusting for prognostic covariates through multivariable regression will help mitigate selection bias due to dependent censoring [31]. Additionally, the relative effect from the Cox model was converted into a measure of impact by combining the HR with cancer-free survival data obtained from the analysis of the BMS non-recipient cohort. We also compared the naïve (disregarding immortal time), PTDM, and ITDM methods to evaluate differences in time alignment and their effects on the estimated association between BMS and cancer risk.

To assess whether the study data aligned with a population model hypothesizing no effect, a *P* value was calculated. The exact *P* value indicates the probability of the estimated effect (or more extreme), had the tested hypothesis been the source of the study data. Results yielding *P* values <0.05 were classified as unusual (statistically significant) given the tested hypothesis [32]. For clinical benefit assessment, the point estimate and its 95% confidence interval (CI) were reported, delineating the range of test hypotheses within which the study data would fall within the central 95% of the distribution specified in these models [32]. All analyses were conducted using Stata (version 18; Stata Corp, College Station, TX, USA).

## Results

### Cohort characteristics

**Figure 1.**
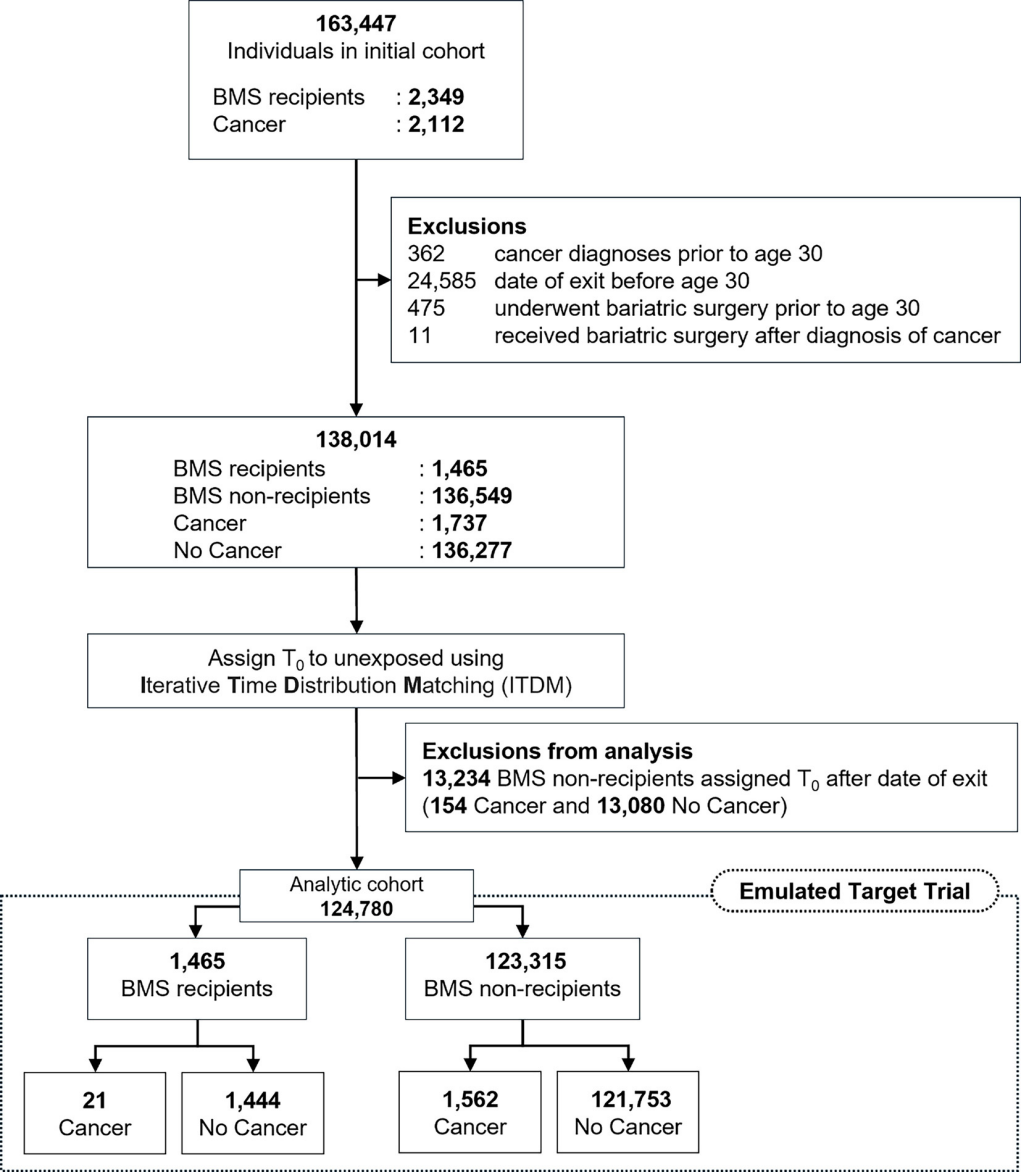
**Cohort selection and emulation of target trial**. Abbreviation: BMS: Bariatric metabolic surgery.

As illustrated in Figure 1, we identified 163,447 adults with more than one documented BMI ≥30 prior to age 40. Among this initial cohort, 2349 individuals had undergone BMS, and 2112 had received a cancer diagnosis. In our dataset, 81.7% (*n* ═ 1921) of surgery recipients underwent sleeve gastrectomy, while Roux-en-Y gastric bypass accounted for 18.2% (*n* ═ 428). Cancer cases encompassed various malignancies coded according to ICD-10AM nomenclature, with the most prevalent being thyroid cancer (C73, *n* ═ 461), breast cancer (C50, *n* ═ 392), and colorectal cancer (C18, *n* ═ 60). The complete distribution of cancer types is detailed in Table S2.

The incidence of cancer among individuals without BMS varied over the follow-up period post age 30. In the first five years, the incidence rate was 0.96 (95% CI: 0.89–1.05) per 1000 person-years, increasing to 2.58 (95% CI: 2.41–2.77) in the subsequent five years. The rate further escalated to 3.13 (95% CI: 2.80–3.50) in the following five years and peaked at 6.75 (95% CI: 4.52–10.07) beyond 15 years of follow-up. Kaplan–Meier survival estimates indicated a corresponding decline in cancer-free survival probability, decreasing from 99.5% (95% CI: 99.46%–99.55%) at five years to 98.2% (95% CI: 98.08%–98.28%) at ten years, and 96.4% (95% CI: 96.15%–96.66%) at 15 years. Gender-specific rates are presented in Table 1.

**Table 1:** Cancer incidence and cancer free survival without BMS and impact of BMS

**Period in years from age 30y**	**Cancers**	**Person-years (per 1000)**	**Cancer incidence (per 1000 person-years)**	**Cancer free survival**	NNT*
*Males*					
0–5	215	227.5	0.94 (0.83, 1.08)	At 5y: 99.5%	409
5–10	294	137.1	2.14 (1.91, 2.40)	At 10y: 98.4%	125
10–15	100	42.2	2.37 (1.95, 2.89)	At 15y: 97.1%	69
15+	5	1.5	3.43 (1.43, 8.25)		
*Females*					
0–5	340	348.8	0.97 (0.87, 1.08)	At 5y: 99.5%	393
5–10	541	186.0	2.90 (2.67, 3.17)	At 10y: 98%	98
10–15	202	54.5	3.71 (3.23, 4.26)	At 15y: 96%	48
15+	19	2.1	9.05 (5.77, 14.18)		
*Final cox PH model results*					
Hazard ratio	95% CI	z	*P>|z|*		
0.49	0.31, 0.76	--3.16	0.002		

*Based on an HR of 0.49; NNT, number needed to treat to prevent one additional case of cancer, is assessed over follow-up periods of 5, 10, and 15 years. **Adjusted for gender, Qatari nationality, type 2 diabetes at baseline, class III obesity (BMI ≥ 40 kg/m^2^ or not) at origin, age at study entry, and smoking status. Abbreviations: BMS: Bariatric metabolic surgery; NNT: Number needed to treat; HR: Hazard ratio; PH: Proportional hazards; CI: Confidence interval.

To establish eligibility at age 30, exclusions were applied sequentially. Individuals diagnosed with cancer before the study’s date of origin were excluded (*n* ═ 362), as were those whose follow-up ended prior to this date (*n* ═ 24,585). Participants who underwent BMS before age 30 (*n* ═ 475) and those who had BMS after a cancer diagnosis (*n* ═ 11) were also excluded. Following these exclusions, the refined cohort comprised 138,014 individuals available for analysis, including 1465 individuals who underwent BMS and 1737 diagnosed with cancer.

### Assignment of T_0_

As outlined in the analysis plan, we employed the ITDM approach for T_0_ assignment in individuals who did not receive BMS (unexposed). The selection of optimal iterations was based on two complementary methods: (1) a quantitative evaluation of the alignment of immortal-time distributions across the two comparison groups, utilizing the Kolmogorov–Smirnov D statistic—a distributional difference measure that is independent of sample size—and (2) a visual examination of the corresponding distribution plots. In this analysis, iteration 6 exhibited the lowest D value and demonstrated the best overlap between the groups in the distribution plots. The ITDM Transition Plot and the distribution plots from multiple iterations are included as supplementary material (see Figures S1 and S2). Consequently, 13,234 BMS non-recipients who had T_0_ after exiting the risk set were excluded from the analysis. This process resulted in the removal of 529 cancer cases, comprising 148 blood cancers, 128 thyroid cancers, 60 breast cancers, 43 reproductive system cancers, 28 gastrointestinal cancers, and 122 cases of other malignancies. These exclusions were essential to maintain the alignment of follow-up time. A detailed breakdown of the excluded cancer cases is provided in Table S2. The final analysis cohort comprised 124,780 individuals, with 1583 failures (a reduction from the initial 1737) in a single-failure-per-subject dataset.

### Description of the analysis cohort

The final analysis cohort consisted of 124,780 individuals, among whom 1465 (1.17%) were BMS recipients. The median age at entry (age at T_0_) was 35.87 years (IQR: 33.25–38.63). Women represented 61.17% (*n* ═ 76,331) of the cohort and 69.90% (*n* ═ 1,024) of BMS recipients. Qataris made up 20.67% (*n* ═ 25,795) of the overall cohort, while accounting for 86.08% (*n* ═ 1261) of BMS recipients. Class III obesity (BMI ≥ 40 kg/m^2^) was present in 11.13% (*n* ═ 13,893) of the cohort, with a significantly higher prevalence among BMS recipients (56.93%, *n* ═ 834). The median follow-up duration was 7.79 years (IQR: 4.89–10.85). Overall, cancer was diagnosed in 1.27% (*n* ═ 1583) of individuals, with a slightly higher incidence among BMS recipients (1.43%, *n* ═ 21). This latter comparison is a naïve crude assessment, as it includes immortal time. Comprehensive characteristics of the cohort are detailed in Table 2.

**Table 2:** Characteristics of individuals included in the final analysis dataset

	**BMS recipients (*n* ═ 1465)**	**BMS non-recipients (*n* ═ 123,315)**	**SMD**	**Overall (*n* ═ 124,780)**
*Demographics*				
Age at entry (T_0_), years (median, IQR)	35.63 (32.74–38.72)	35.88 (33.25–38.63)	0.004	35.87 (33.25–38.63)
Female (*n*, %)	1024 (69.90%)	75,307 (61.07%)	0.187	76,331 (61.17%)
Qatari (*n*, %)	1,261 (86.08%)	24,534 (19.9%)	1.771	25,795 (20.67%)
*Obesity-related comorbidities at baseline*				
Class III Obesity - BMI ≥ 40 kg/m^2^ (*n*, %)	834 (56.93%)	13,059 (10.57%)	1.124	13,893 (11.13%)
Type 2 diabetes (*n*, %)	40 (2.73%)	1642 (1.33%)	0.099	1682 (1.35%)
Hypertension (*n*, %)	30 (2.05%)	1072 (0.87%)	0.098	1102 (0.88%)
Dyslipidemia (*n*, %)	22 (1.50%)	1102 (0.89%)	0.056	1124 (0.90%)
*Smoking status*				
Current smoker (*n*, %)	172 (11.74%)	10,004 (8.11%)	0.068	10,176 (8.16%)
Non-smoker (*n*, %)	633 (43.21%)	49,141 (39.85%)	0.122	49,774 (39.89%)
Missing/unknown (*n*, %)	660 (45.05%)	64,170 (52.04%)	--0.140	64,830 (51.96%)
Follow-up, years (median, IQR)	10.09 (6.69–12.90)	7.77 (4.88–10.82)	0.461	7.79 (4.89–10.85)
Cancer cases (*n*, %)	21 (1.43%)	1,562 (1.27%)	0.014	1583 (1.27%)

Abbreviations: BMS: Bariatric metabolic surgery; SMD: Standardized mean difference; IQR: Interquartile range; BMI: Body mass index.

### Cox regression analysis

The stratified (for gender and smoking status) Cox regression analysis, after adjusting for other confounders and prognostic covariates, indicated a statistically significant benefit of surgery (HR, 0.49; 95% CI, 0.31–0.76; *P* ═ 0.002), which is also of practical importance. The E-value was 3.50 for the point estimate and 1.96 for the upper CI bound. The HR translated to numbers needed to treat (NNTs) of 409, 125, and 69 for males, and 393, 98, and 48 for females at 5, 10, and 15 years of follow-up, respectively. Thus, at 15 years of follow-up, one less cancer was observed for every 69 surgically vs non-surgically managed males with obesity, and for every 48 surgically vs non-surgically managed females with obesity (see Table 1). Model diagnostics confirmed adequate fit, with the global goodness-of-fit plot (Figure S4) showing a close correspondence between observed and expected cumulative hazard functions. To evaluate proportionality, a stratified proportional-hazards test (stphtest) was conducted. Stratification by gender and smoking status yielded a global PH test (χ^2^ ═ 7.15, *P* ═ 0.209), indicating acceptable proportionality. The log–log survival curves (Figure S5) revealed broadly similar patterns for surgery status and other covariates throughout most of the follow-up period, with divergence occurring only at the extremes, suggesting approximate adherence to the proportional-hazards assumption for the primary exposure. A sensitivity analysis was performed to evaluate the impact of the limited number of cancer cases among surgical recipients (*n* ═ 21). Repeating the analysis multiple times while randomly removing two BMS recipients with cancer showed no significant effect on the estimates, indicating robustness to potential outcome misclassification (see Table S3).

### Comparison of three methods

**Figure 2.**
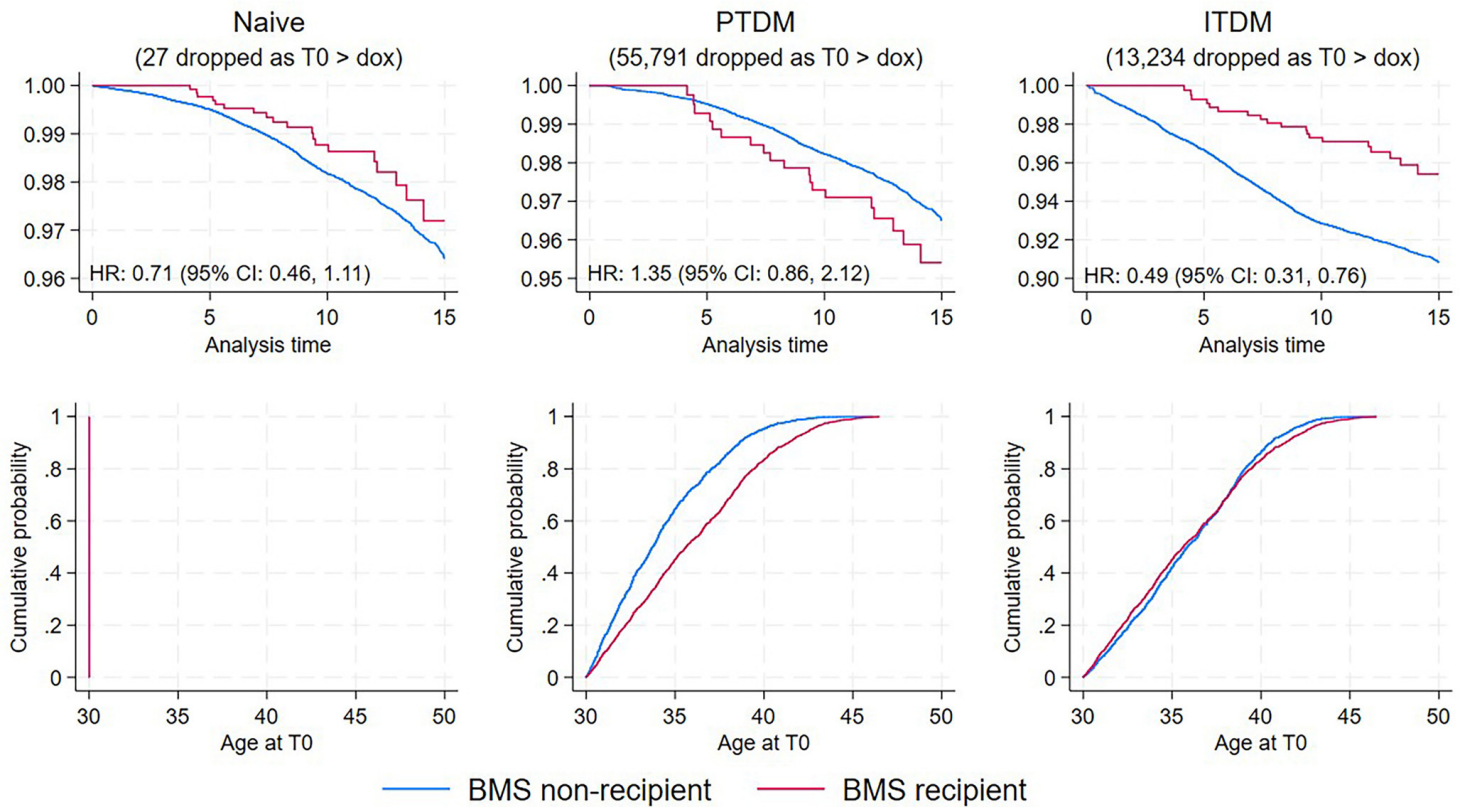
**Comparison of three analytical approaches.** The top row illustrates unadjusted survival curves, while the bottom row displays the distribution of assigned immortal time across groups (with no immortal time assigned in the left-most panels). The left panel shows 1737 failures, the middle panel shows 978 failures, and the right panel shows 1583 failures observed in single-failure-per-subject data. All individuals who were excluded (failures) were members of the BMS non-recipient group. Abbreviations: BMS: Bariatric metabolic surgery; PTDM: Prescription time distribution matching; ITDM: Iterative time distribution matching; HR: Hazard ratio; CI: Confidence interval; dox: Date of exit from the study.

Figure 2 illustrates how misalignment of immortal time impacts the comparison between the two groups in survival analysis. The top panel of Figure 2 demonstrates that the alignment of survival curves is contingent upon the accurate assignment of T_0_. The naïve analysis neglects immortal time. The PTDM fully corrects the misalignment of T_0_ but assigns a substantial number of T_0_ beyond the participants’ exit date from the study, resulting in worse misalignment than the naïve method due to exclusions (see the middle panel of Figure 2). Conversely, the ITDM minimizes misalignment by avoiding participant dropouts, thereby preserving the failure pool (1583 failures) and accurately aligning immortal time. This is illustrated by the distribution plot in the right panel. The failures observed in single-failure-per-subject data amounted to 1737, 978, and 1583 for the naïve, PTDM, and ITDM methods, respectively, indicating that the ITDM not only minimized immortal time misalignment but also significantly reduced exclusions. All individuals with failures who were excluded were BMS non-recipients.

## Discussion

Obesity is a chronic and life-threatening condition closely linked to an elevated risk of various cancers. Annually, approximately 5% of new cancer cases in men and 10% in women are attributable to excess body weight, highlighting the significant global burden of obesity-related malignancies [33]. The International Agency for Research on Cancer (IARC) has identified 13 cancers for which there is substantial evidence that excess adiposity is a causal factor, including prevalent malignancies such as colorectal, thyroid, and postmenopausal breast cancer [34]. The mechanisms underlying this relationship are complex and likely vary by cancer type, with chronic inflammation, dysregulated immunity, hyperinsulinemia, and changes in sex hormone metabolism all potentially contributing factors [2, 35]. In this context, our study provides new evidence regarding the impact of BMS on subsequent cancer development. We found a significantly lower cancer risk among individuals who underwent BMS, with a HR of 0.49 (95% CI, 0.31, 0.76) in our synchronized Cox proportional hazards model, adjusted for confounders and prognostic covariates. These results indicate a statistically significant effect (*P* ═ 0.002), suggesting a strong protective benefit against cancer risk for BMS recipients.

Among surgical procedures, Roux-en-Y gastric bypass has been reported to offer greater benefits compared to laparoscopic sleeve gastrectomy [36]. However, our analysis could not draw similar comparisons due to the limited number of cancer cases following BMS. This observation may reflect the degree of weight loss associated with each procedure. Notably, some studies suggest that gastric bypass may be linked to an increased risk of colorectal cancer [11, 13, 37], while others indicate a potential reduction in colorectal cancer incidence [8, 38], or present non-significant estimates that either favor benefits [21, 39] or indicate harm [40]. Due to the limited number of incident cancers among BMS recipients in our study, we could not rigorously test this hypothesis. Of the 41 cancers identified in BMS recipients, only 2 (5%) were gastrointestinal, compared to 139 of 2071 cancers (7%) in non-recipients, suggesting no meaningful difference descriptively. Within our dataset, 65% of gastrointestinal cancers (92/141) were colorectal cancers. Additionally, concerns have been raised regarding a potential association between sleeve gastrectomy and esophageal cancer due to its effects on gastroesophageal reflux disease (GERD) and Barrett’s esophagus [41]. Recent studies have reported a lower incidence of Barrett’s esophagus post-sleeve gastrectomy, with no significant differences in reflux esophagitis rates between sleeve gastrectomy and gastric bypass [42]. Our dataset included only one case of esophageal cancer, rendering direct comparisons impractical.

A significant concern in observational studies of this nature is immortal time bias. Immortal time bias arises when follow-up time for specific groups is misclassified, often by excluding periods during which events cannot occur. This bias is commonly observed in cohort comparisons between exposed and unexposed individuals. If the distribution of time between age 30 and T_0_ differs between BMS recipients and non-recipients, it could artificially skew risk estimates, with the direction of bias contingent on the nature of the follow-up time misalignment, as illustrated in Figure 2. The discrepancies in follow-up time across groups in existing observational studies help explain the variability in results concerning the incidence of all cancers following BMS [8, 9, 21, 39, 40, 43–45]. The first panel in Figure 2 illustrates the approach most observational studies have taken, yielding an adjusted HR of 0.70 (95% CI 0.45,1.09), which aligns with findings from existing literature and is biased toward the null. While many studies did not explicitly address immortal time bias in their design or analysis [46], one study by Lazzati et al. [21] made an effort to do so. The authors reported following all subjects from their initial diagnosis of obesity to cancer diagnosis, death, or censoring. However, since the assignment to BMS occurred after the follow-up commenced, their study likely still experienced misclassification of follow-up time, potentially introducing bias. In our study, we explicitly addressed the misclassification of follow-up time.

A notable strength of our approach is its transparent strategy for mitigating immortal time bias. This methodology, in contrast to others, employs time alignment that minimally alters the target population, and the estimator does not rely on unrealistic functional form assumptions [47]. The iterative process further enhances this approach by systematically refining T_0_ assignment in the non-surgery group until the distribution of immortal time aligns optimally with that of the surgery group. This alignment prevents the introduction of artificial lead time and survival advantages, thereby improving the internal validity of the study. The extent of distortion in the magnitude of reported associations due to continued misclassification of pre-exposure person time is contingent upon three factors [48]: a) the mean pre-exposure (immortal) time, b) the proportion of exposed individuals, and c) the length of follow-up in the study. The bias is exacerbated as the immortal time and proportion of exposed participants increase, while it diminishes with longer follow-up periods. The impact of the immortal time factor observed in this study is vividly illustrated in Figure 2.

Despite the strengths of our approach, several limitations must be acknowledged. First, this study remains observational, and methodologies such as ITDM cannot fully eliminate potential biases. Second, while target trial emulation addresses issues related to improper design, it does not resolve limitations associated with data quality [49]. Despite our efforts to ensure data accuracy, our sources may contain errors in BMI measurements, dates, or cancer diagnoses due to reliance on electronic medical record systems. For instance, smoking status was inadequately captured, with over half the cohort lacking this information, which hinders effective adjustment. Third, although our DAG depicts the minimal adjustment set that may reduce bias from dependent censoring, not all relevant variables may have been included, leaving the potential for residual bias. Notably, nationality was accurately recorded and included in the adjustment set, which should mitigate bias from this source (see the DAG in Figure S3); however, because the DAG does not account for unmeasured variables, residual and unaccounted confounding remain possible. Fourth, the relatively shorter follow-up for some participants and the limited number of cancer outcomes following BMS should be considered when interpreting these results, as they restrict procedure-specific analyses. Most non-recipients of BMS were residents with shorter follow-up periods compared to citizens. Fifth, data on post-operative weight loss, BMI changes, or metabolic remission were unavailable in our dataset. Nonetheless, these variables likely serve as mediators of the effect of BMS on cancer risk, and their absence is unlikely to materially affect the direction and magnitude of our estimates (see DAG in Figure S3). It is important to note that these modeling assumptions, based on the DAG, are explicitly stated; other researchers may adopt different modeling assumptions that they find acceptable. Cancer risk reduction following BMS is believed to stem from weight loss, hormonal changes, or both, with recent evidence suggesting a predominant hormonal effect, making our estimates likely conservative if some participants did not meet these targets.

A notable strength of this study is our assessment of risk for all cancers combined, which provides a comprehensive estimate of the total disease burden associated with an exposure. By pooling all cancer types, we increase the number of cases, thereby enhancing statistical power and reducing random variation, while also circumventing the multiple comparisons problem inherent in analyzing specific cancer types separately. This approach can reveal a net increase in cancer risk that may be overlooked when examining individual cancers, which may show only modest or inconsistent associations. Additionally, it serves as an early indicator of potential carcinogenicity warranting further site-specific investigation. However, all-cancer analyses may obscure differing site-specific effects, where an exposure could elevate risk for some cancers while reducing it for others. Therefore, these analyses should be complemented with future type-specific investigations to provide a complete risk profile.

## Conclusion

This study offers preliminary evidence that BMS is associated with a reduced hazard of cancer in a national EHR cohort utilizing target trial emulation with ITDM. Given the unavailability of post-operative outcomes and the possibility of residual confounding, this association should be interpreted with caution. Larger datasets with procedure-specific outcomes and more comprehensive covariate capture are necessary to refine effect estimates and examine site-specific risks. By integrating the emulation of a target trial approach, we enhance our capacity to draw credible inferences from observational data. Future examinations of the ITDM approach by other researchers will ensure that target trial emulation methods continue to evolve, and the simplicity of this approach may enhance its impact on epidemiologic research, thereby enabling real-world evidence to more confidently inform clinical decision-making.

## Supplemental data

Supplemental data are available at the following link: https://www.bjbms.org/ojs/index.php/bjbms/article/view/12842/4040.

## Data Availability

The dataset generated and analyzed during this study is available from the corresponding author upon reasonable request.

## References

[1] Powell-Wiley TM, Poirier P, Burke LE, Després JP, Gordon-Larsen P, Lavie CJ (2021). Obesity and cardiovascular disease: a scientific statement from the American Heart Association. Circulation.

[2] Wolin KY, Carson K, Colditz GA (2010). Obesity and cancer. Oncologist.

[3] Wiebe N, Stenvinkel P, Tonelli M (2019). Associations of chronic inflammation, insulin resistance, and severe obesity with mortality, myocardial infarction, cancer, and chronic pulmonary disease. JAMA Netw Open.

[4] Gregor MF, Hotamisligil GS (2011). Inflammatory mechanisms in obesity. Annu Rev Immunol.

[5] Arnold M, Pandeya N, Byrnes G, Renehan AG, Stevens GA, Ezzati M (2015). Global burden of cancer attributable to high body-mass index in 2012: a population-based study. Lancet Oncol.

[6] Cancer attributable to obesity [Internet]. https://gco.iarc.fr/causes/obesity/tools-pie.

[7] Lim P-W, Stucky C-CH, Wasif N, Etzioni DA, Harold KL, Madura JA (2024). Bariatric surgery and longitudinal cancer risk: a review. JAMA Surg.

[8] Schauer DP, Feigelson HS, Koebnick C, Caan B, Weinmann S, Leonard AC (2019). Bariatric surgery and the risk of cancer in a large multisite cohort. Ann Surg.

[9] Sjöström L, Gummesson A, Sjöström CD, Narbro K, Peltonen M, Wedel H (2009). Effects of bariatric surgery on cancer incidence in obese patients in Sweden (Swedish Obese Subjects Study): a prospective, controlled intervention trial. Lancet Oncol.

[10] Gerber P, Naqqar D, von Euler-Chelpin M, Kauppila JH, Santoni G, Holmberg D (2024). Incidence of cancer and cardiovascular disease after bariatric surgery in older patients. JAMA Netw Open.

[11] Ostlund MP, Lu Y, Lagergren J (2010). Risk of obesity-related cancer after obesity surgery in a population-based cohort study. Ann Surg.

[12] Ashrafian H, Ahmed K, Rowland SP, Patel VM, Gooderham NJ, Holmes E (2011). Metabolic surgery and cancer: protective effects of bariatric procedures. Cancer.

[13] Derogar M, Hull MA, Kant P, Östlund M, Lu Y, Lagergren J (2013). Increased risk of colorectal cancer after obesity surgery. Ann Surg.

[14] De Roover A, Detry O, Desaive C, Maweja S, Coimbra C, Honoré P (2006). Risk of upper gastrointestinal cancer after bariatric operations. Obes Surg.

[15] Scozzari G, Trapani R, Toppino M, Morino M (2013). Esophagogastric cancer after bariatric surgery: systematic review of the literature. Surg Obes Relat Dis Off J Am Soc Bariatr Surg.

[16] Doumouras AG, Lovrics O, Paterson JM, Sutradhar R, Paszat L, Sivapathasundaram B (2023). Residual risk of breast cancer after bariatric surgery. JAMA Surg.

[17] Sjöholm K, Andersson-Assarsson JC, Kristensson FM, Hjorth S, Garelius HG, Jacobson P (2023). Long-term incidence of haematological cancer after bariatric surgery or usual care in the Swedish Obese Subjects study: a prospective cohort study. Lancet Healthy Longev.

[18] Tee MC, Cao Y, Warnock GL, Hu FB, Chavarro JE (2013). Effect of bariatric surgery on oncologic outcomes: a systematic review and meta-analysis. Surg Endosc.

[19] Kim MS, Kim JY, Song YS, Hong S, Won HH, Kim WJ (2024). Association of bariatric surgery with indicated and unintended outcomes: an umbrella review and meta-analysis for risk-benefit assessment. Obes Rev Off J Int Assoc Study Obes.

[20] Courcoulas AP, Daigle CR, Arterburn DE (2023). Long term outcomes of metabolic/bariatric surgery in adults. BMJ.

[21] Lazzati A, Epaud S, Ortala M, Katsahian S, Lanoy E (2022). Effect of bariatric surgery on cancer risk: results from an emulated target trial using population-based data. Br J Surg.

[22] Madenci AL, Kurgansky KE, Dickerman BA, Gerlovin H, Wanis KN, Smith AD (2024). Estimating the effect of bariatric surgery on cardiovascular events using observational data?. Epidemiol Camb Mass.

[23] Rubino F, Cummings DE, Eckel RH, Cohen RV, Wilding JP, Brown WA (2025). Definition and diagnostic criteria of clinical obesity. Lancet Diabetes Endocrinol.

[24] Hernán MA, Robins JM (2016). Using big data to emulate a target trial when a randomized trial is not available. Am J Epidemiol.

[25] Karim ME, Petkau J, Gustafson P, Platt RW, Tremlett H, BeAMS Study Group. (2018). Comparison of statistical approaches dealing with time-dependent confounding in drug effectiveness studies. Stat Methods Med Res.

[26] Zhou Z, Rahme E, Abrahamowicz M, Pilote L (2005). Survival bias associated with time-to-treatment initiation in drug effectiveness evaluation: a comparison of methods. Am J Epidemiol.

[27] Karim ME, Gustafson P, Petkau J, Tremlett H (2016). Comparison of statistical approaches for dealing with immortal time bias in drug effectiveness studies. Am J Epidemiol.

[28] Pencina MJ, Larson MG, D’Agostino RB (2007). Choice of time scale and its effect on significance of predictors in longitudinal studies. Stat Med.

[29] AbdulMajeed J, Khatib M, Dulli M, Sioufi S, Al-Khulaifi A, Stone J (2024). Use of conditional estimates of effect in cancer epidemiology: an application to lung cancer treatment. Cancer Epidemiol.

[30] Hernán MA, Hernández-Díaz S (2012). Beyond the intention to treat in comparative effectiveness research. Clin Trials Lond Engl.

[31] Xuan J, Mt-Isa S, Latimer N, Bell Gorrod H, Malbecq W, Vandormael K (2025). Is inverse probability of censoring weighting a safer choice than per-protocol analysis in clinical trials?. Stat Methods Med Res.

[32] Zar LA, Abdulmajeed J, Elshoeibi AM, Syed A, Awaisu A, Glasziou P (2025). From significance to divergence: guiding statistical interpretation through language. Curr Opin Epidemiol Public Health.

[33] Islami F, Goding Sauer A, Gapstur SM, Jemal A (2019). Proportion of cancer cases attributable to excess body weight by US state, 2011-2015. JAMA Oncol.

[34] Lauby-Secretan B, Scoccianti C, Loomis D, Grosse Y, Bianchini F, Straif K (2016). Body fatness and cancer—Viewpoint of the IARC working group. N Engl J Med.

[35] Kolb R, Sutterwala FS, Zhang W (2016). Obesity and cancer: inflammation bridges the two. Curr Opin Pharmacol.

[36] Tsui ST, Yang J, Zhang X, Spaniolas K, Kim S, Griffin T (2021). The risk of female-specific cancer after bariatric surgery in the state of New York. Surg Endosc.

[37] Mackenzie H, Markar SR, Askari A, Faiz O, Hull M, Purkayastha S (2018). Obesity surgery and risk of cancer. Br J Surg.

[38] Khalid SI, Maasarani S, Wiegmann J, Wiegmann AL, Becerra AZ, Omotosho P (2022). Association of bariatric surgery and risk of cancer in patients with morbid obesity. Ann Surg.

[39] Adams TD, Stroup AM, Gress RE, Adams KF, Calle EE, Smith SC (2009). Cancer incidence and mortality after gastric bypass surgery. Obesity.

[40] Tao W, Santoni G, Von Euler-Chelpin M, Ljung R, Lynge E, Pukkala E (2020). Cancer risk after bariatric surgery in a cohort study from the five Nordic countries. Obes Surg.

[41] Jaruvongvanich V, Matar R, Ravi K, Murad MH, Vantanasiri K, Wongjarupong N (2020). Esophageal pathophysiologic changes and adenocarcinoma after bariatric surgery: a systematic review and meta-analysis. Clin Transl Gastroenterol.

[42] Leslie D, Wise E, Sheka A, Abdelwahab H, Irey R, Benner A (2021). Gastroesophageal reflux disease outcomes after vertical sleeve gastrectomy and gastric bypass. Ann Surg.

[43] Rustgi VK, Li Y, Gupta K, Minacapelli CD, Bhurwal A, Catalano C (2021). Bariatric surgery reduces cancer risk in adults with nonalcoholic fatty liver disease and severe obesity. Gastroenterology.

[44] Anveden Å, Taube M, Peltonen M, Jacobson P, Andersson-Assarsson JC, Sjöholm K (2017). Long-term incidence of female-specific cancer after bariatric surgery or usual care in the Swedish Obese Subjects Study. Gynecol Oncol.

[45] Aminian A, Wilson R, Al-Kurd A, Tu C, Milinovich A, Kroh M (2022). Association of bariatric surgery with cancer risk and mortality in adults with obesity. JAMA.

[46] Suissa K, Schneeweiss S, Glynn RJ, Wexler DJ, Suissa S, Paik JM (2024). Bariatric surgery and all-cause mortality: a methodological review of studies using a non-surgical comparator. Diabetes Obes Metab.

[47] Dang LE, Balzer LB (2023). Start with the target trial protocol, then follow the roadmap for causal inference. Epidemiology.

[48] Harding BN, Weiss NS (2019). Point: immortal time bias—what are the determinants of its magnitude?. Am J Epidemiol.

[49] Hernán MA, Dahabreh IJ, Dickerman BA, Swanson SA (2025). The target trial framework for causal inference from observational data: why and when is it helpful?. Ann Intern Med.

